# Broadband diffuse optical spectroscopy of two-layered scattering media containing oxyhemoglobin, deoxyhemoglobin, water, and lipids

**DOI:** 10.1142/s1793545822500201

**Published:** 2022-04-07

**Authors:** Giles Blaney, Martina Bottoni, Angelo Sassaroli, Cristianne Fernandez, Sergio Fantini

**Affiliations:** * Department of Biomedical Engineering, Tufts University 4 Colby St, Medford, MA 02155, USA; † Department of Biomedical Engineering, Politecnico di Torino 24 Corso Duca degli Abruzzi, Torino, TO 10129, Italy

**Keywords:** Broadband spectroscopy, two-layer medium, heterogeneous forward model, homogeneous inverse model, partial-volume effect

## Abstract

We investigated the relationship between chromophore concentrations in two-layered scattering media and the apparent chromophore concentrations measured with broadband optical spectroscopy in conjunction with commonly used homogeneous medium inverse models. We used diffusion theory togenerate optical data from a two-layered distribution of relevant tissue absorbers, namely, oxyhemoglobin, deoxyhemoglobin, water, and lipids, with a top-layer thickness in the range 1–15 mm. The generated data consisted of broadband continuous-wave (CW) diffuse reflectance in the wavelength range 650–1024 nm, and frequency-domain (FD) diffuse reflectance at 690 and 830 nm; two source-detector distances of 25 and 35 mm were used to simulate a dual-slope technique. The data were inverted using diffusion theory for a semi-infinite homogeneous medium to generate reduced scattering coefficients at 690 and 830 nm (from FD data) and effective absorption spectra in the range 650–1024 nm (from CW data). The absorption spectra were then converted into effective total concentration and oxygen saturation of hemoglobin, as well as water and lipid concentrations. For absolute values, it was found that the effective hemoglobin parameters are typically representative of the bottom layer, whereas water and lipid represent some average of the respective concentrations in the two layers. For concentration changes, lipid showed a significant cross-talk with other absorber concentrations, thus indicating that lipid dynamics obtained in these conditions may not be reliable. These systematic simulations of broadband spectroscopy of two-layered media provide guidance on how to interpret effective optical properties measured with similar instrumental setups under the assumption of medium homogeneity.

## 1. Introduction

Biomedical diffuse optics typically utilizes a simplified model to analyze data collected from a complex medium. That complex medium being biological tissue, which has a heterogeneous distribution of optical properties, and that simplified model often being the semi-infinite homogeneous model.^1^ Therefore, recovered optical properties are said to be effective homogeneous properties. This meaning that a homogeneous medium with these properties would replicate the behavior of the data. When the measurement of a single region is sought, such as the brain,^2^ muscle,^3^ or breast lesions,^4^ these heterogeneous effects on homogeneous recovered data are considered partial-volume confounds.^5^ Therefore, an understanding of how heterogeneous properties are related to effective homogeneous properties may help with data interpretation.

Simulations of optically diffuse media offer a method for generating forward model data where the actual optical properties within the medium are known and may be varied. This allows one to generate forward data from a complex medium and then invert the data using a homogeneous model as one would with experimental data, to compare the actual simulated medium to the recovered values. Highly complex heterogeneous media may be simulated with Monte Carlo methods. These methods may simulate voxelized or meshed media with computationally efficient parallel computations of photon propagation.^6^ Monte Carlo simulations have the advantage of being able to simulate complex media but these methods do not match analytical expressions in terms of speed. Due to how fast analytical expressions may be evaluated, many media of differing properties can be simulated in a short computation time by repeating the analytical evaluation. However, the media simulated by analytical methods must be simpler compared to Monte Carlo. It may be said that the simplest heterogeneous medium is the two-layer medium for which analytical expressions for the diffuse reflectance exist.^7^ In this work, analytical expressions from diffusion theory for the diffuse reflectance in a two-layer medium are used to generate forward data and expressions for a semi-infinite homogeneous medium are used to invert data.

The optical parameters describing these media can be split into two categories, those affecting the (*μ*_*a*_) and those affecting the $\left(\mu_{s}^{\prime }\right)$.^8^ Absorption parameters are related to chromophore concentration. Considering biological tissue in the Near-InfraRed (NIR) range, the primary chromophores are Oxy-hemoglobin (*O*), Deoxy-hemoglobin (*D*), Water (*W*), and Lipid (*L*). In this work this notation of one letter symbols is used to represent concentrations of these chromophores. Scattering on the other hand can be described by a power law containing two parameters, an amplitude^a^ and the scattering power law exponent (*b*).

NIR Spectroscopy (NIRS), whether Continuous-Wave (CW), Frequency-Domain (FD), or Time-Domain (TD), seeks to measure *μ*_*a*_ and $\mu_{s}^{\prime }$, or combinations of the two, in the NIR range with the ultimate goal of recovering chromophore concentrations, scattering parameters, or both. These measurements are conducted at either discrete or broadband NIR optical wavelengths (*λs*), with a general trade-off of temporal information for spectral information.^9^ Broadband spectroscopy creates a overdetermined system when recovering chromophores, allowing for better understanding of how the chromophore model fits the data.^11^ When considering broadband absorption, the *O* and *D* parameters can be re-expressed as Total-hemoglobin (*T*) and oxygen Saturation (*S*), where *T* = *O* + *D* and *S* = *O*/*T*, which have more spectral meaning^b^ as amplitude and shape, respectively.

This work is an extension of a previous investigation utilizing Dual-Slope (DS) broadband diffuse optics on human muscle tissue.^11^ This previous work observed the aforementioned complex interaction between a heterogeneous tissue and the recovered parameters, and attempted to apply a two-layer model to the measured data. As an extension of that work, this work utilizes a systematic set of simulations to obtain a deeper understanding of how properties in a two-layer medium affect the recovered values for the instruments and methods in the previous work.^11^ Despite the results herein being specific to the simulated instrument and recovery methods, this methodology of careful examination of a two-layer biological medium may provide broader guidance in the field of biomedical diffuse optics.

## 2. Methods

### 2.1. Simulated instrumental conditions

A specific measurement system was simulated when generating forward data. Thus, this measurement system or instrument was not used in the real-world for this work. The real-world version of the simulated instrument was implemented^10^ and utilized for tissue measurements^11^ in the past. Only instrument parameters pertinent to the simulations are described here.

The simulated instrument was comprised of both a FD and CW portion. The FD portion measured complex reflectance phasors $\left({\tilde{R}}_{\text{s}}\right)$ with a modulation frequency of 140.625 MHz at two discrete *λs* of 690 and 830 nm. The CW portion measured real reflectances (*Rs*) at a broadband range of wavelengths (650–1024 nm spaced by 0.5 nm). Both the FD and CW reflectances were simulated at two source-detector distances (*ρs*) of 25 and 35 mm. Therefore, the simulated instrument outputs: four FD reflectance phasors ($\tilde{R}(\lambda ,\rho )$; two *λs* and two *ρs*) and 1498 CW reflectances (*R*(*λ*, *ρ*); 749 *λs* and two *ρs*).

The real-life version of the instrument relied on a DS optode arrangement to eliminate the need for calibration^12^ and provide preferentially deep sensitivity.^13,14^ The calibration consideration is not pertinent for these simulations, however the expected preferentially deep sensitivity of DS does apply to the simulated measurements.

### 2.2. Two-layer forward model

The forward model used expressions derived from diffusion theory to simulated a two-layer medium and retrieve diffuse reflectance both $\tilde{R}$ and *R*. The input of the model described chromophore concentrations and reduced scattering parameters in the two layers as well as the thickness, these 13 parameters are as follows: top layer Total-hemoglobin (*T*_Top_), bottom layer Total-hemoglobin (*T*_Bottom_), top layer oxygen Saturation (*S*_Top_), bottom layer oxygen Saturation (*S*_Bottom_), top layer Water (*W*_Top_), bottom layer Water (*W*_Bottm_), top layer Lipid (*L*_Top_), bottom layer Lipid (*L*_Bottom_), top layer reduced scattering coefficient at 830 nm $\left(\mu_{s,\text{Top}}^{\prime }(830\;\text{nm})\right)$, bottom layer reduced scattering coefficient at 830 nm $\left(\mu_{s,\text{Bottom}}^{\prime }(830\;\text{nm})\right)$, top layer scattering power law exponent (*b*_Top_), bottom layer scattering power law exponent (*b*_Bottom_), and top layer thickness (*Z*_Top_).

The first step of the model was to calculate the *μ*_*a*_ spectrum^c^ for each layer from the absorption parameters: *T*_Top_, *T*_Bottom_, *S*_Top_, *S*_Bottom_, *W*_Top_, *W*_Bottom_, *L*_Top_, and *L*_Bottom_. These layer-dependent absorption spectra were calculated as a linear combination of known extinction coefficients (*ϵ*s)^15–17^ weighted by chromophore concentrations (*C*s):

(1) $${\vec{\mu }}_{a}=E\vec{C}$$

(2) $$\left[\begin{array}{c} \mu_{a}\left(\lambda_{1}\right)\\ \mu_{a}\left(\lambda_{2}\right)\\ \vdots \end{array}\right]=\left[\begin{array}{cccc} \epsilon_{O}\left(\lambda_{1}\right) & \epsilon_{D}\left(\lambda_{1}\right) & \epsilon_{W}\left(\lambda_{1}\right) & \epsilon_{L}\left(\lambda_{1}\right)\\ \epsilon_{O}\left(\lambda_{2}\right) & \epsilon_{D}\left(\lambda_{2}\right) & \epsilon_{W}\left(\lambda_{2}\right) & \epsilon_{L}\left(\lambda_{2}\right)\\ \vdots & \vdots & \vdots & \vdots \end{array}\right]\left[\begin{array}{c} ST\\ (1-S)T\\ W\\ L \end{array}\right],$$

where the *ϵ*s are contained within matrix of extinction coefficients (**E**), $\vec{C}$ is the vector of chromophore concentrations, *O* = *ST*, and *D* = (1 − *S*)*T*.

Next, the $\mu_{s}^{\prime }$ spectrum was calculated for each layer using a power law:

(3) $$\mu_{s}^{\prime }(\lambda )=\mu_{s}^{\prime }(830\;\text{nm}){\left(\frac{\lambda }{830\;\text{nm}}\right)}^{-b},$$

for each layer using the scattering parameters: $\mu_{s,\text{Top}}^{\prime }(830\;\text{nm})$, $\mu_{s,\text{Bottom}}^{\prime }(830\;\text{nm})$, *b*_Top_, and *b*_Bottom_.

The analytical two-layer model took the *μ*_*a*_ and $\mu_{s}^{\prime }$ spectra for each layer and *z*_Top_ as input with a final unmentioned parameter of index of refraction (n) which was assumed to be fixed at 1.4 for both layers. This analytical two-layer model has been previously derived^7^ and utilized.^11^ Here, the retrieval of either $\tilde{R}$ or *R* is simulated given the above input parameters for each *ρ* and *λ*. The expression for the two-layer diffuse reflectance can be found in Appendix A.1 (Eq. (A.23)).

### 2.3. Semi-infinite homogeneous inverse model

Recovery of absolute optical properties was achieved using an iterative linear fit of a linearized reflectance versus *ρ*. Expressions for this linearized reflectance were derived from diffusion theory for the semi-infinite homogeneous medium. This method was previously described^10,11^ and is explained in Appendix A.2. Using this method, $\tilde{R}$ recovered complex effective attenuation coefficient $\left({\tilde{\mu }}_{\text{eff}}\right)$, from which *μ*_*a*_ and $\mu_{s}^{\prime }$ were obtained:

(4) $$\mu_{t}^{\prime }\left(\lambda_{\text{FD}}\right)=\frac{-2cℜ\left[{\tilde{\mu }}_{\text{eff}}\left(\lambda_{\text{FD}}\right)\right]ℑ\left[{\tilde{\mu }}_{\text{eff}}\left(\lambda_{\text{FD}}\right)\right]}{3n\omega },$$

(5) $$\mu_{a}\left(\lambda_{\text{FD}}\right)=\frac{{ℜ}^{2}\left[{\tilde{\mu }}_{\text{eff}}\left(\lambda_{\text{FD}}\right)\right]-{ℑ}^{2}\left[{\tilde{\mu }}_{\text{eff}}\left(\lambda_{\text{FD}}\right)\right]}{3\mu_{t}^{\prime }\left(\lambda_{\text{FD}}\right)},$$

(6) $$\mu_{s}^{\prime }\left(\lambda_{\text{FD}}\right)=\mu_{t}^{\prime }\left(\lambda_{\text{FD}}\right)-\mu_{a}\left(\lambda_{\text{FD}}\right),$$

using the parameters of speed of light in vacuum (*c*), *n*, angular modulation frequency (*ω*), and total reduced attenuation coefficient $\left(\mu_{t}^{\prime }\right)$. The FD subscript signifies that the property was recovered for two *λs*.

For broadband measurements the FD properties must be extrapolated. In this method, the FD data was only used for $\mu_{s}^{\prime }$, thus $\mu_{s}^{\prime }$ at two *λs* was extrapolated by rearranging Eq. (3):

(7) $$b_{\text{rec}}=\frac{\text{ln}\left(\mu_{s}^{\prime }(830\;\text{nm})/\mu_{s}^{\prime }(690\;\text{nm})\right)}{\text{ln}(690\;\text{nm}/830\;\text{nm})}.$$

A continuum of extrapolated $\mu_{s}^{\prime }$ was then be calculated across all CW *λs* using this recovered *b* and $\mu_{s}^{\prime }$ at 830 nm recovered by FD by inputting these parameters into Eq. (3).

Next, the simulated CW *R* was used to recover broadband effective attenuation coefficient (*μ*_eff_) using the aforementioned iterative recovery methods^10,11^ (Appendix A.2). This was combined with the extrapolated $\mu_{s}^{\prime }$^d^ to give a broadband measurement of *μ*_*a*_:

(8) $$\mu_{a}\left(\lambda_{\text{CW}}\right)=\sqrt{\frac{\mu_{s}^{\prime 2}\left(\lambda_{\text{CW}}\right)}{4}+\frac{\mu_{\text{eff}}^{2}\left(\lambda_{\text{CW}}\right)}{3}}-\frac{\mu_{s}^{\prime }\left(\lambda_{\text{CW}}\right)}{2},$$

where the CW subscript signifies that a value is calculated for each of the 749 CW *λs*.

Finally, the recovered CW *μ*_*a*_ is converted to effective homogeneous^e^ recovered *T*, *S*, *W*, and *L* using the MATLAB (MathWorks, Natick, MA, USA) mldivide function to invert Eq. (1). Since the **E** is nonsquare, MATLAB’s qr decomposition is used:

(9) $$\text{E}=\text{Q}\text{R}=\left[\begin{array}{c} {\text{Q}}_{1}\;{\text{Q}}_{2} \end{array}\right]\left[\begin{array}{c} {\text{R}}_{1}\\ 0 \end{array}\right],$$

where **Q**_1_ and **Q**_2_ have orthogonal columns and **R**_1_ is an upper triangular matrix. From this decomposition Eq. (1) can be inverted on the recovered *μ*_*a*_ spectrum:

(10) $$\vec{C}=R_{1}^{-1}\left({Q_{1}}^{T}{\vec{\mu }}_{a}\left(\lambda_{\text{CW}}\right)\right),$$

giving the effective homogeneous recovered chromophores.

Therefore, the output of this inverse model necessary to describe an equivalent homogeneous medium is two scattering parameters and four absorption parameters. The scattering parameters are the effective homogeneous recovered $\mu_{s}^{\prime }$ at one *λ*^f^ and the effective homogeneous recovered *b*. The absorption parameters are the effective homogeneous recovered *T*, *S*, *W*, and *L*.

### 2.4. Two-layer sensitivity

Sensitivities to changes in absorption parameters in each layer of a two-layer medium were also considered. These sensitivities were calculated as numerical derivatives, where an independent variable^g^ was changed by a small amount^h^ and the dependent variable’s^i^ change was retrieved. The ratio of the change in the dependent variable divided by the independent variable was considered the sensitivity of the dependent variable to a change in the independent variable. In all cases $\mu_{s}^{\prime }$ recovered from FD was fixed at the baseline value when recovering the small change in the dependent variable. Thus only changes in absorption parameters were considered, and importantly cross-talk between absorption parameters and scattering parameters was ignored. In practice this would be equivalent to making only one FD measurement at the beginning of an experiment and then measuring absorption dynamics with only CW, therefore assuming constant scattering during an experiment.

The aforementioned independent absorption variables and inputs to the forward model (Sec. 2.2) were *T*_Top_, *T*_Bottom_, *S*_Top_, *S*_Bottom_, *W*_Top_, *W*_Bottom_, *L*_Top_, and *L*_Bottom_. The dependent absorption variables were the recovered *T*, *S*, *W*, and *L*. Sensitivities could take the form of any pair between these independent and dependent variables. These sensitivities were split into two categories, first co-sensitivities which are between like parameters^j^ and second cross-sensitivities which are between differing parameters.^k^

Taking the example of the co-sensitivity between *T*_Top_ and *T* (*δT*/*δT*_top_), this should be interpreted as how much a change in the top-layer *T* will affect the effective homogeneous recovered *T*. These sensitivity values also are affected by the baseline properties, therefore this dependence was also investigated by recalculating sensitivities for various values of baseline two-layer absorption parameters.

## 3. Results

### 3.1. Recovery of absolute values

The results in this section focus on how actual properties in a two-layer medium are related to the absolute recovered effective homogeneous properties. This was done by simulating the $\tilde{R}$ and *R* for a two layer medium (Sec. 2.2) with the particular parameters of the simulated instrument (Sec. 2.1), then inverting these data using a semi-infinite homogeneous model (Sec. 2.3).

#### 3.1.1. Baseline model

Table 1 shows the baseline absorption model and the recovered baseline absorption properties for said model. Table 2 shows scattering values for this same baseline model. Unsurprisingly, in this case the recovered parameters take values between the actual values of the two layers. However, the dominant layer is different for different parameters, for example recovered *T* is closer to the actual bottom-layer value but recovered *W* is approximately the average of the top and bottom layers.

#### 3.1.2. Variation of model parameters

Starting with the baseline two-layer model parameters stated in Tables 1 and 2, each parameter was individually varied.^l^ For every new set of two-layer forward model absorption and scattering properties the recovered values were then found.

**Fig. 1.**
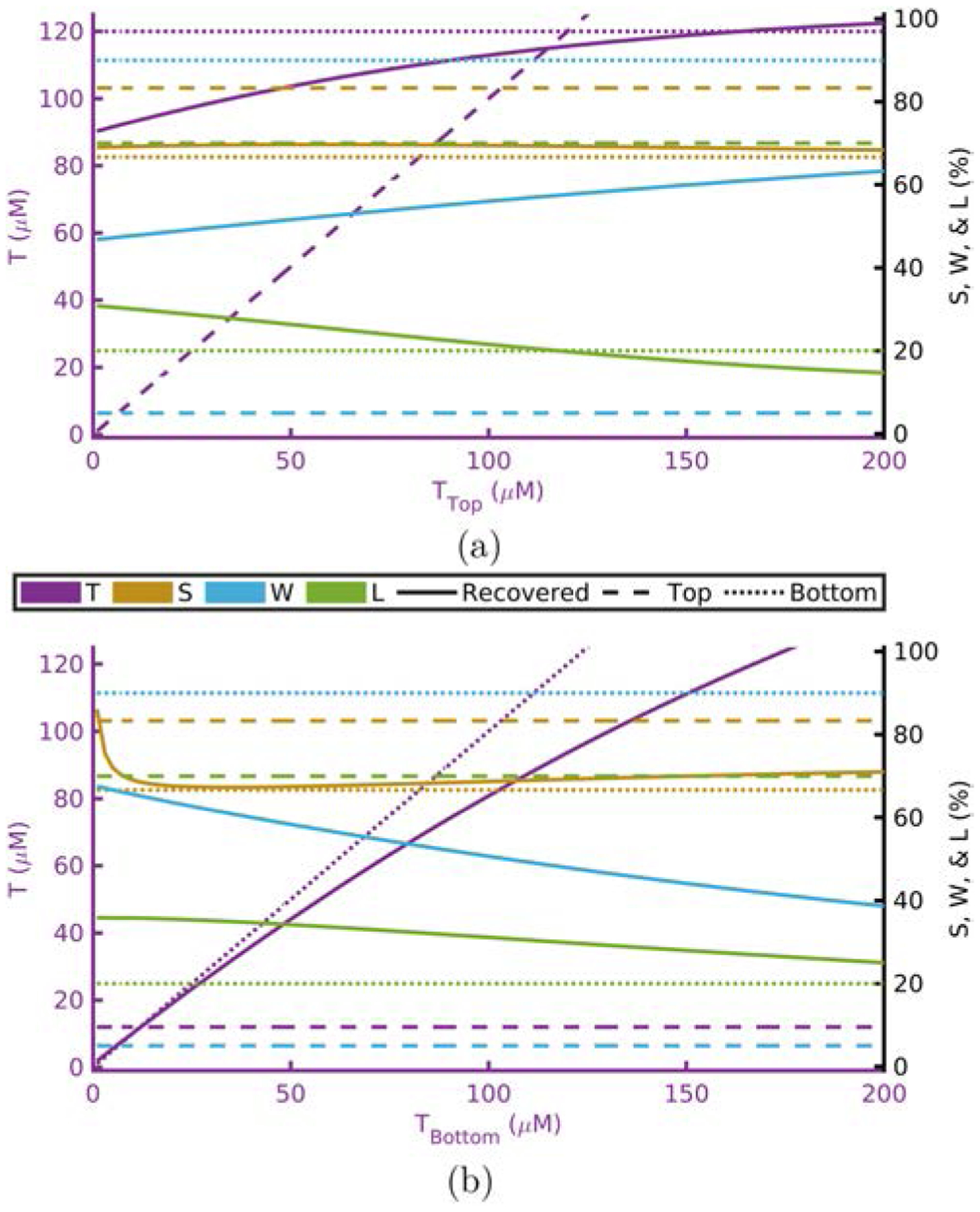
Recovered values versus actual top layer (a) or bottom layer (b) *T* fixing others to Tables 1 and 2. Acronyms: Total-hemoglobin (*T*), oxygen Saturation (*S*), Water (*W*), and Lipid (*L*).

**Fig. 2.**
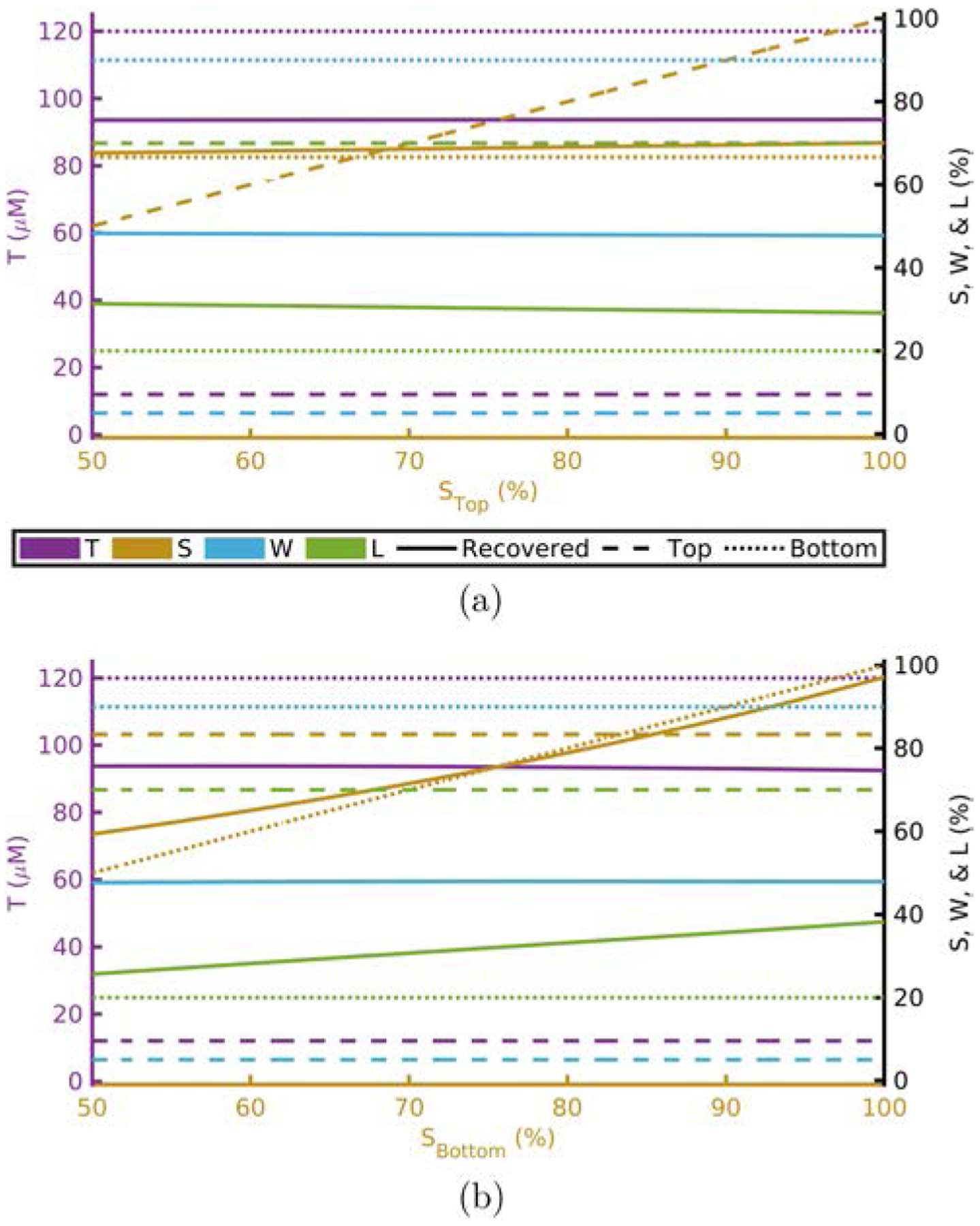
Recovered values versus actual top layer (a) or bottom layer (b) *S* fixing others to Tables 1 and 2. Acronyms: Total-hemoglobin (*T*), oxygen Saturation (*S*), Water (*W*), and Lipid (*L*).

**Fig. 3.**
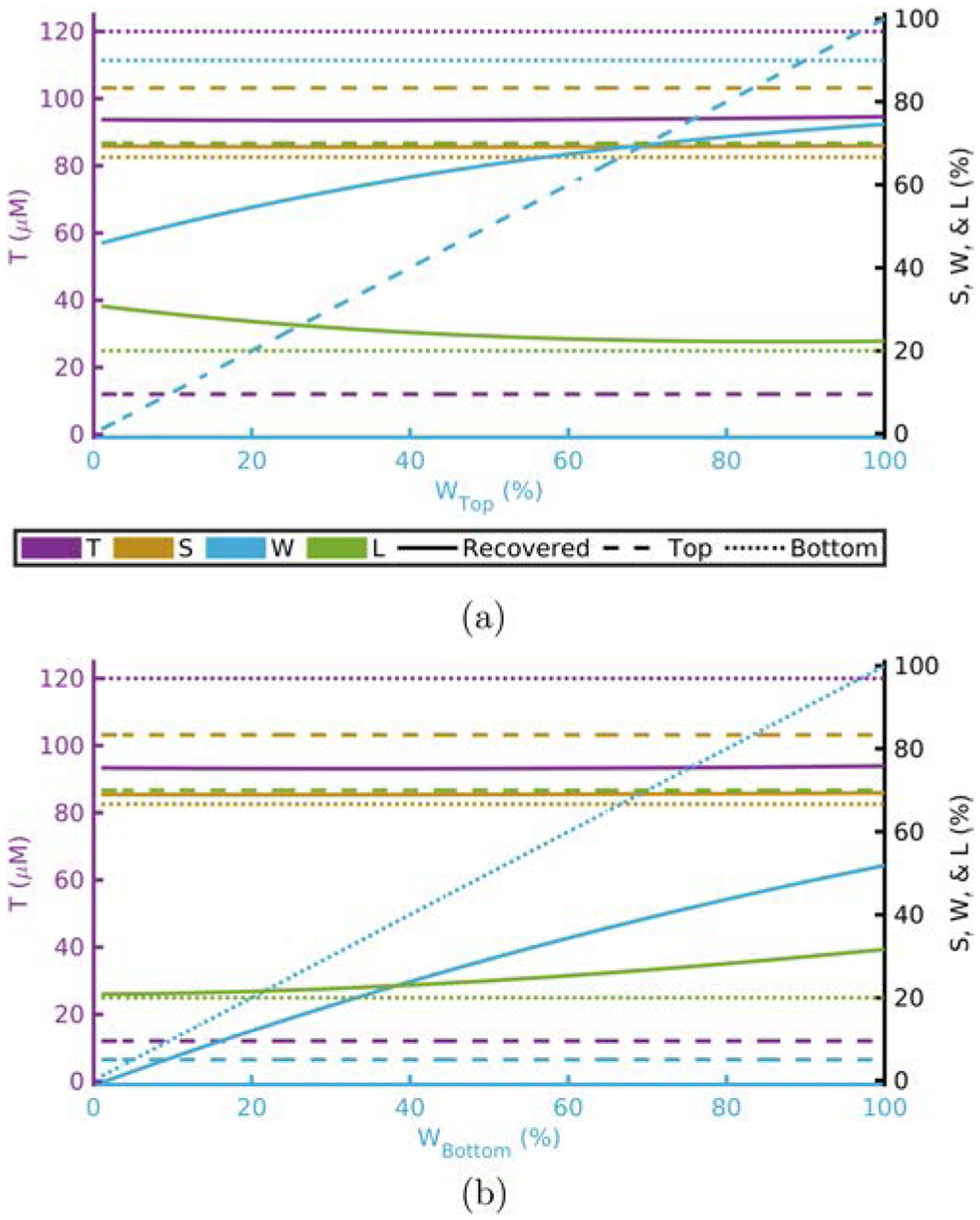
Recovered values versus actual top layer (a) or bottom layer (b) *W* fixing others to Tables 1 and 2. Acronyms: Total-hemoglobin (*T*), oxygen Saturation (*S*), Water (*W*), and Lipid (*L*).

**Fig. 4.**
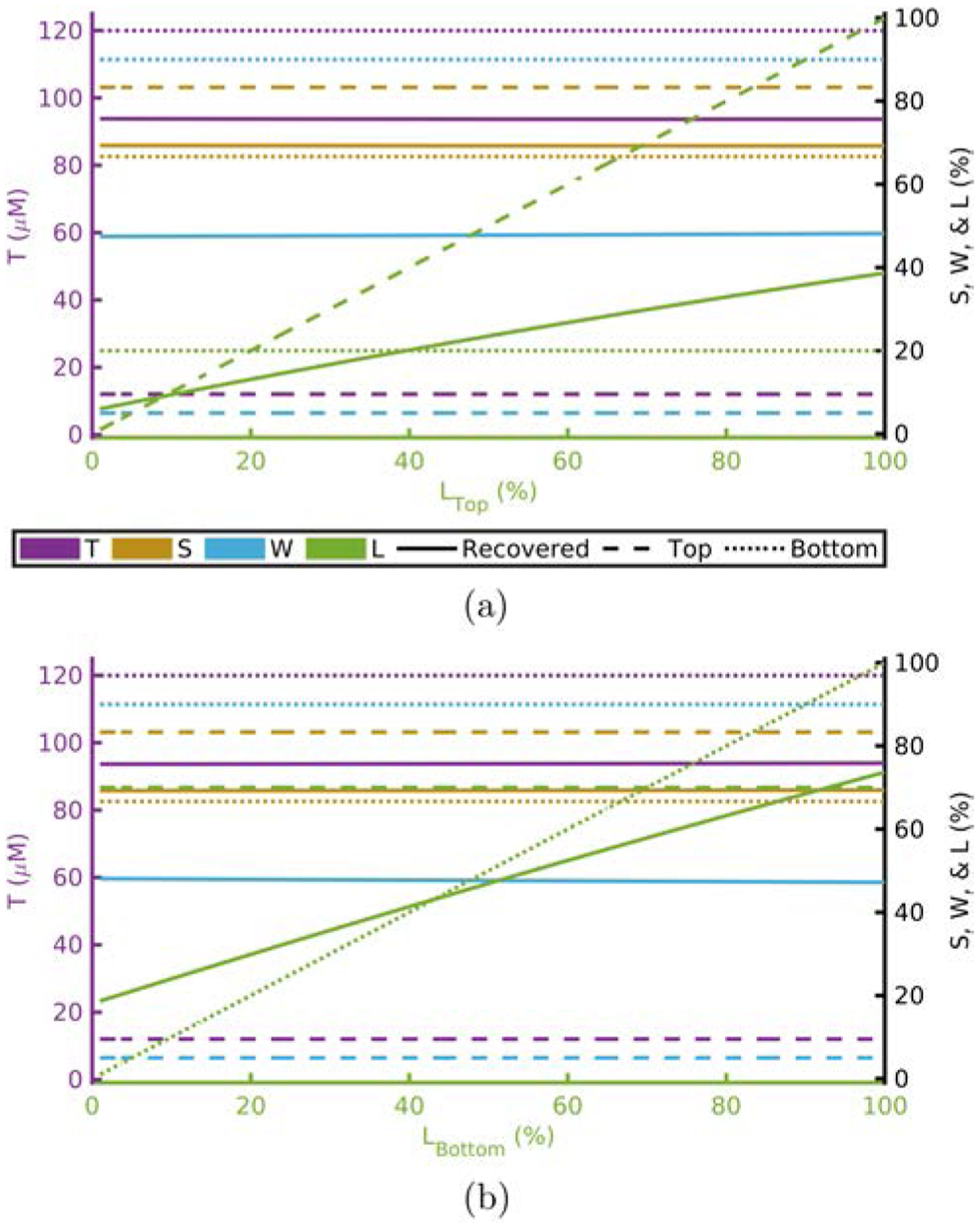
Recovered values versus actual top layer (a) or bottom layer (b) *L* fixing others to Tables 1 and 2. Acronyms: Total-hemoglobin (*T*), oxygen Saturation (*S*), Water (*W*), and Lipid (*L*).

Figures 1–4 show this computational experiment for the absorption parameters with Figs. 1–4 varying *T*, *S*, *W*, and *L*, respectively. In each of these, the variation of the top-layer parameter is shown in subplot (a) and the variation of the bottom layer in (b). Solid lines show the recovered value of the parameter while dashed or dotted lines show the actual top or bottom parameter values, respectively. By examining these plots one may determine how close a recovered parameter is to the actual value in a particular layer^m^ as a function of one of the absorption parameters. For example, Fig. 1(a) shows that the recovered value for *T* is always closer to *T*_Bottom_ regardless of the value of *T*_Top_.^n^

**Fig. 5.**
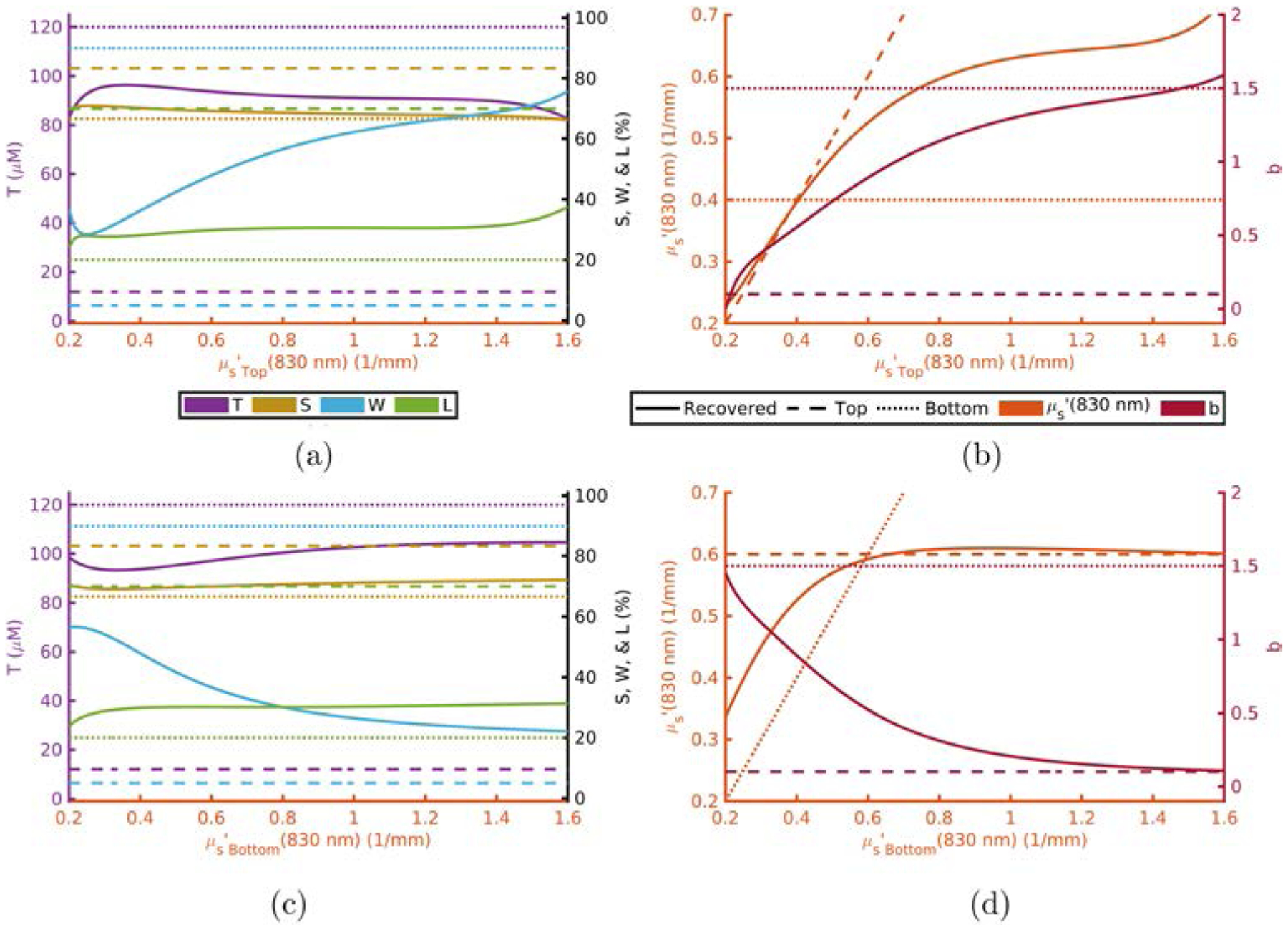
Absorption (a), (c) and scattering (b), (d) recovered values versus actual top layer (a), (b) or bottom layer (c), (d) $\mu_{s}^{\prime }$ fixing others to Tables 1 and 2. Acronyms: Total-hemoglobin (*T*), oxygen Saturation (*S*), Water (*W*), Lipid (*L*), reduced scattering coefficient $\left(\mu_{s}^{\prime }\right)$ and scattering power law exponent (*b*).

**Fig. 6.**
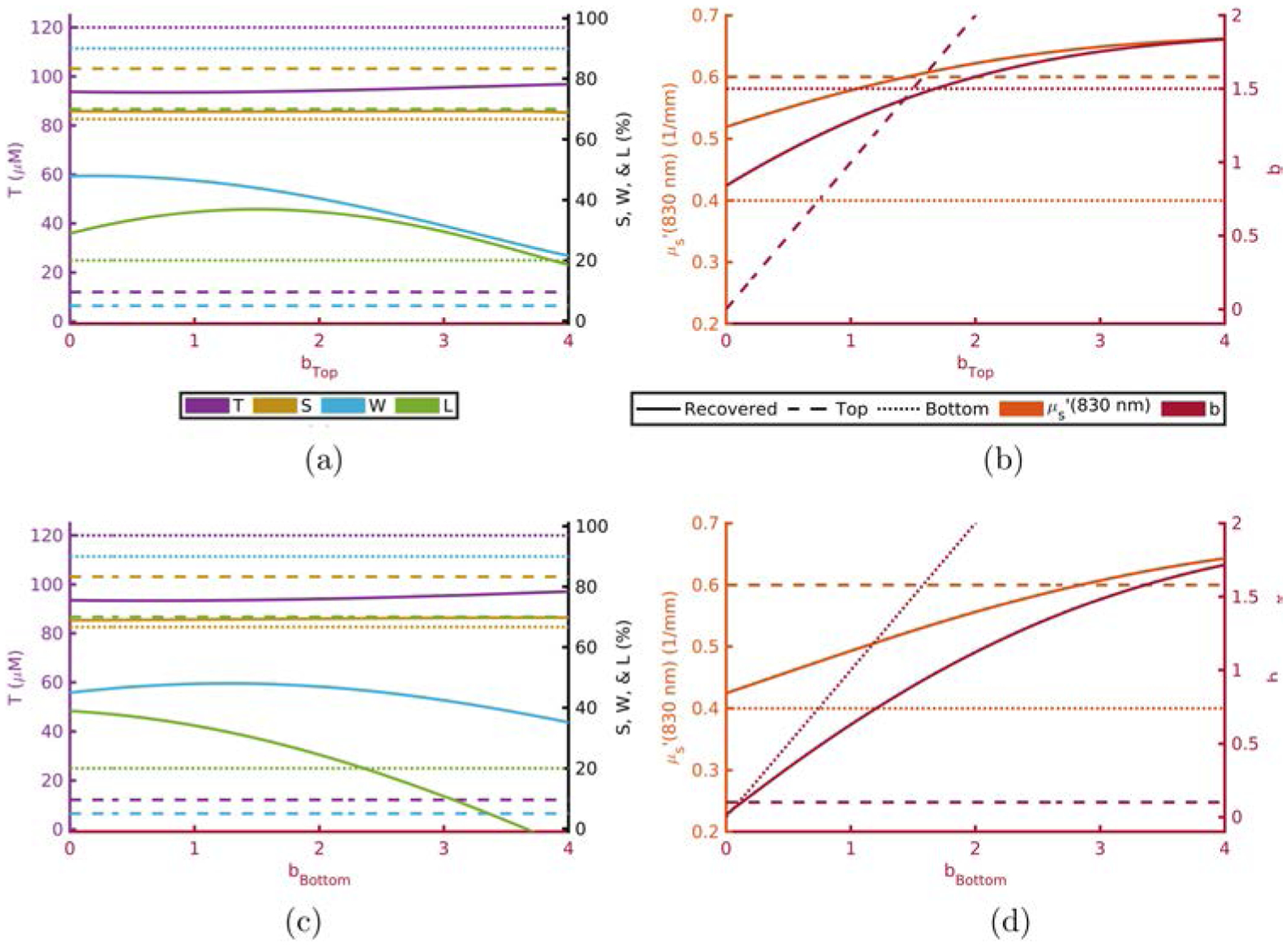
Absorption (a), (c) and scattering (b), (d) recovered values versus actual top layer (a), (b) or bottom layer (c), (d) *b* fixing others to Tables 1 and 2. Acronyms: Total-hemoglobin (*T*), oxygen Saturation (*S*), Water (*W*), Lipid (*L*), reduced scattering coefficient $\left(\mu_{s}^{\prime }\right)$ and scattering power law exponent (*b*).

Scattering parameters have a somewhat more complex effect on the recovered values. This is seen in Figs. 5 and 6 for $\mu_{s}^{\prime }$ and *b*, respectively. These figures display the recovered absorption parameters while varying top layer (a) or bottom layer (c) scattering properties as well as the recovered scattering parameters while varying top layer (b) or bottom layer (d) scattering properties. An example of the aforementioned complex effects can be seen in Fig. 5(b) where the recovered value of *b* may take values not in-between *b*_Top_ and *b*_Bottom_ for large values of $\mu_{s,\text{Top}}^{\prime }(830\;\text{nm})$.

**Fig. 7.**
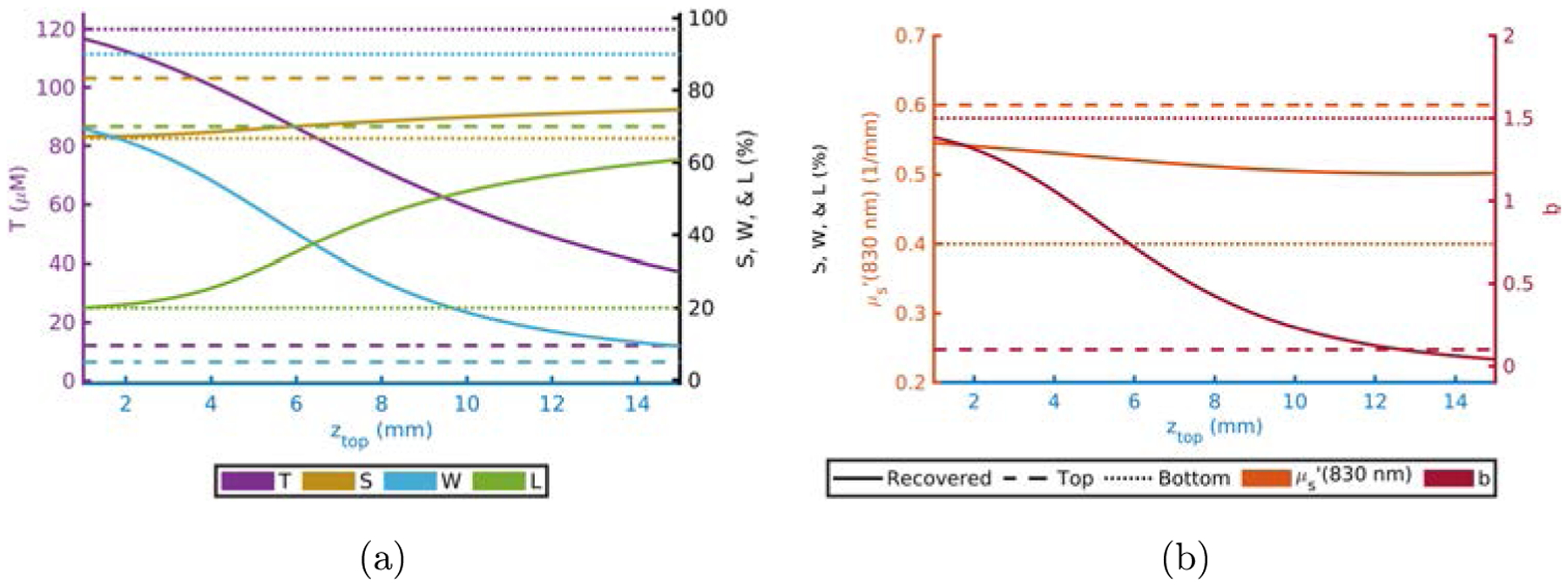
Absorption (a) and scattering (b) recovered values versus *z*_Top_ fixing others to Tables 1 and 2. Acronyms: Total-hemoglobin (*T*), oxygen Saturation (*S*), Water (*W*), Lipid (*L*), reduced scattering coefficient $\left(\mu_{s}^{\prime }\right)$ scattering power law exponent (*b*) and top layer thickness (*z*_Top_).

Finally, Fig. 7 displays the results from the computational experiment where *z*_Top_ was varied. In this case, the recovered parameters can be seen to start closer to the actual values for the bottom layer when *z*_Top_ is thin and move closer to the actual top-layer value as *z*_Top_ increases, with the curves following a sigmoid like shape. Some interesting results include the dominance of *S*_Bottom_ and $\mu_{s,\text{Top}}^{\prime }(830\;\text{nm})$ on the recovered value even for thick *z*_Top_, considering that end value of *z*_Top_ in the plots is deeper than most of the sensitivity region for these optical measurements.^14^

### 3.2. Sensitivity

The results in this section focus on how changes in absorption parameters in a two-layer medium are related to the recovered effective homogeneous absorption changes.^o^ This was done by calculating sensitivities according to Sec. 2.4.

#### 3.2.1. Baseline model sensitivities

Using the values in Tables 1 and 2 as baseline, co-sensitivities^p^ were calculated (Table 3). From this it can be seen that recovered dynamic changes in *T*, *L*, and especially *S* are more sensitive to bottom-layer dynamics of the like chromophore. Conversely, recovered *W* dynamics are more sensitive to changes in the top-layer. Here it is emphasized that these values are only strictly actual for the particular baseline model chosen, and this consideration will be addressed in the following section.

Table 4 shows the full set of sensitivities displayed as fractions for the baseline model. In this case the co-sensitivities (Table 3) can be found in the diagonal of the upper and lower sub-tables and all off-diagonal elements are cross-sensitivities^q^ which can be thought of as cross-talk between parameters. Examining the cross-sensitivities in Table 4, it appears that there is little cross-talk between parameters, with the only parameter displaying notable cross-talking being the recovered changes in *L*. In the case of measuring recovered *L* dynamics, its value may be affected by changes in *W*_Top_, *S*_Bottom_, and *W*_Bottom_. Again, all values are valid only for the particular baseline model. However, since little cross-sensitivity was observed^r^ overall, the following section will neglect cross-sensitivity for brevity.

#### 3.2.2. Variation sensitivities as a result of varying model parameters

In this section the two-layer absolute properties are varied and co-sensitivities calculated allowing for insight about how these sensitivities are affected by the absolute values of the parameters in a two-layer medium. In all cases parameters were not co-varied and unvaried parameters were held at the values of the baseline model (Tables 1 and 2), simply stated, only one parameter was varied at a time.

**Fig. 8.**
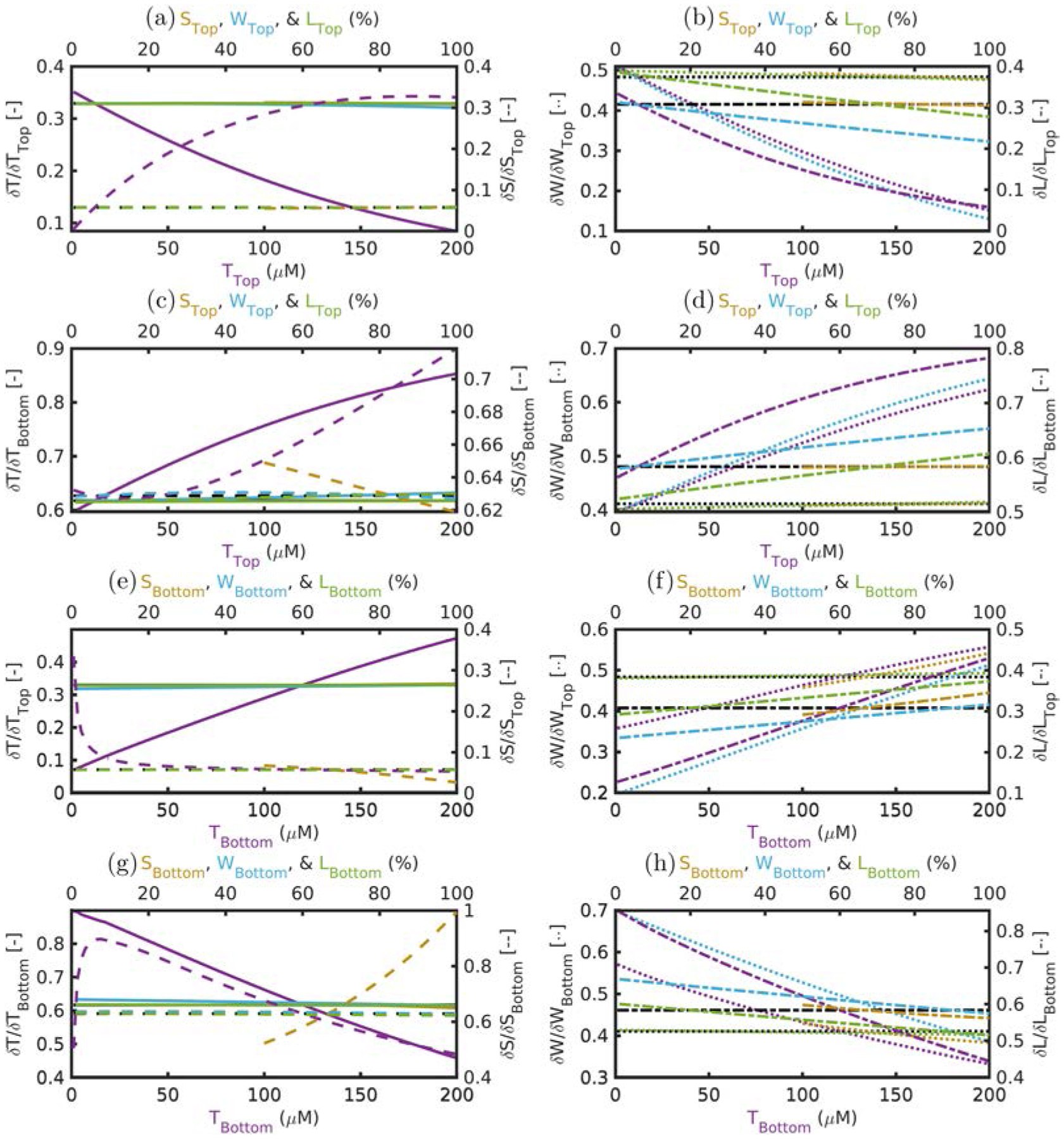
Co-sensitivities of recovered absorption parameters to actual changes in the top layer or bottom layer as a function of the absolute value of actual two-layer absorption parameters. (a), (c), (e), (g) *T* and *S* sensitivities. (b), (d), (f), (h) *W* and *L* sensitivities. (a), (b) Top-layer sensitivities versus top-layer absolute values. (c), (d) Bottom-layer sensitivities versus top-layer absolute values. (e), (f) Top-layer sensitivities versus bottom-layer absolute values. (g), (h) Bottom-layer sensitivities versus bottom-layer absolute values. Absolute values were varied without co-variation, and baseline model parameters are found in Tables 1 and 2, and top layer thickness was 5 mm. Black lines show the co-sensitivities of the baseline model (Table 3). *X*-axes: Curves belong to the *x*-axis of like color. Purple curves correspond to the bottom x-axes and show the variation of the actual absolute value of *T*. Yellow, blue, and green curves correspond to the top *x*-axes and show the variation of the actual absolute value of *S*, *W*, and *L*, respectively. *Y*-axes: Curves belong to the *y*-axis of designated symbol which is shown in square brackets. Solid, dashed, dotted, and dash-dotted lines correspond to sensitivities of *T*, *S*, *W*, and *L*, respectively. Acronyms and variables: Total-hemoglobin (*T*), oxygen Saturation (*S*), Water (*W*), and Lipid (*L*).

These results are displayed in Fig. 8, where each sub-plot contains dual *y*-axes and dual *x*-axes. To which *y*-axis a curve belongs to is designated by the line type that is specified in square brackets of the axis title. All solid lines correspond to *T* sensitivities, dashed lines to *S* sensitivities, dotted lines to *W* sensitivities, and dash-dotted lines to *L* sensitivities. Which *x*-axis a curve belongs to is signified by color, which is the same as the color of the variable in the axis title. All yellow lines show the sensitivity while varying the absolute value of *S*, blue lines while varying the absolute value of *W*, green lines while varying the absolute value of *L*, and purple lines while varying the absolute value of *T*. Each row of subplots examines differing combinations of which layer the sensitivity is to and which layer the absolute value is being varied.

Despite the plethora of information in Fig. 8 the story in most subplots is quite simple. This is because the absolute value of a given parameter does not affect many of the sensitivities in a given subplot. For example, in Fig. 8(a) the sensitivities of recovered *T* or *S* to top-layer changes as a function of actual absolute top-layer properties is plotted. In this case it is immediately obvious that only the purple curves significantly change. This means that the sensitivities of recovered *T* or *S* to top-layer changes are not affected by the absolute value of *S*_Top_, *W*_Top_, or *L*_Top_. However, since the purple line does change, the *T* sensitivity to the top-layer decreases and the *S* sensitivity to the top-layer increases each with greater *T*_Top_.

Examining Fig. 8 as a whole, some general trends are observed. In the left column the curves show that *T* and *S* sensitivities are not affected by the actual absolute values of *W* or *L*, but are somewhat affected by the actual value of *S* and are strongly affected by the actual value of *T*. The right column concerns that sensitivities of *W* and *L* which appear to be more heavily affected by the actual absolute value of all parameters but to a lesser extent on *S*. The actual absolute value of *T* has the most effect on these sensitivities, closely followed by the actual absolute value of *W*. Finally, the actual absolute value of *L* does not affect the *W* sensitivity, but the opposite is not true.

Again, it is noted that all these results are specific to the model in question. Despite the parameters in this experiment being varied, they were not covaried, scattering or thickness were not varied at all, and only the specific measurement method in Sec. 2.1 was considered.

## 4. Discussion

This work sought to investigate how actual properties from a two-layer model are related to the properties retrieved from methods that utilize a DS instrument and semi-infinite homogeneous model at their core. Arguably the two-layer model is the simplest heterogeneous model one can have, and yet its interactions with a homogeneous inversion model are still complex. Examining this simple pair of mismatched forward and inverse models may help provide insight into the meaning of retrieved optical properties from tissues, which are in fact much more complex and heterogeneous than even a two-layer model.

First, the case of recovery of effective absolute homogeneous optical properties was considered (Sec. 3.1). General trends observed in those simulations are:

1. Recovered *T* is dominated by *T*_Bottom_ (Table 1 and Fig. 1(b)).
2. Recovered *S* is dominated by *S*_Bottom_ (Table 1 and Fig. 2).
3. Recovered *W* and *L* are not dominated by either the top- or bottom-layer value alone (Table 1 and Figs. 3 and 4).
4. Actual scattering properties have little effect on recovered *T* and *S* but do effect recovered *W* and *L* (Figs. 5(a), 5(c), 6(a) and 6(c)).
5. Recovered *b* can take values outside of the range between actual *b*_Top_ and *b*_Bottom_ (Fig. 6(b)).
6. Recovered $\mu_{s}^{\prime }$ is dominated by the actual top-layer value (Fig. 7(b)).

As a whole these results provide some valuable insights. For example, a goal in biomedical diffuse optics is to measure the chromophore concentrations of deep tissues. Points (1) and (2) suggest that for this model and measurement method the goal is feasible, at least for a similar *z*_Top_ to the ones studied. Additionally, point (5) may provide some insight into apparent scattering powers that are outside of the range of what is physically possible.^s^ Finally, these results show that each individual recovered property is not representative of the same region as the others. For example, point (6) suggests that recovered $\mu_{s}^{\prime }$ is dominated by the top layer but point (1) suggests that the opposite is true for *T*. Since the recovered $\mu_{s}^{\prime }$ resulted from the FD measurement and *μ*_*a*_ from CW, this may be evidence that FD exhibits more superficial sensitivity compared to CW. This is consistent with the idea that at increasing modulation frequencies sensitivity to optical property changes becomes more superficial.^13^ However, this effect only occurs at frequencies above 500 MHz, therefore it is likely that this is not the effect here. Thus, this observed effect is likely simply due to the $\mu_{s}^{\prime }$ measurement being dominated by superficial layers.

The results for sensitivity (Sec. 3.2) tell a similar story to those of absolute properties, but with the additional focus on dynamic changes in absorption while assuming scattering is constant. Again, a list of general observations follows:

- Recovered dynamics in absorption parameters are significantly affected by each layer except for *S* whose dynamics are dominated by the bottom layer (Table 4).
- There is little cross-talk in dynamics of different parameters except for *L* which has significant cross-talk with *W*_Top_, *S*_Bottom_, and *W*_Bottom_ (Table 4).
- The actual absolute value of *T* or *W* affects the sensitivity of almost every parameter (Fig. 8).
- The actual absolute value of *S* has little effect, for the most part, on the sensitivity of almost every parameter except the sensitivity recovered *S* to *S*_Bottom_ (Figs. 8(c) and 8(g)).
- The actual absolute value of *L* only has significant effect on *L* sensitivity (Figs. 8(b), 8(d), 8(f) and 8(h)).

As may be expected in most cases, sensitivities are affected by the absolute value of multiple parameters within a two-layer medium. However, point (8) may be a significant result. This being that *L* dynamics will have significant cross-talk with *W* and *S* dynamics due to the high cross-sensitivity (Table 4). Therefore, caution is recommended when allowing *L* to dynamically change in analysis, especially for the wavelength range examined here. This confound may not be a significant issue since *L* is not typically expected to dynamically change, thus it is reasonable and, likely safer due to the cross-talk, to assume no *L* changes from baseline during one’s analysis.

These results should be considered when interpreting certain recovered properties or dynamic changes in a heterogeneous medium. Furthermore, since the particular results are dependent on instrumental setup, it may be helpful for one to repeat such simulations for one’s own instrument, thus aiding in the interpretation of data from said instrument. The two-layer medium is much simpler than the complex heterogeneous one within biological tissue, but the model does provide insight into how different instruments and methods behave when measuring heterogeneity.

## 5. Conclusion

Within this article various simulations were carried out to evaluate a particular simulated instrument (Sec. 2.1) and optical property inversion model (Sec. 2.3). The simulations focused on using a two-layer forward data (Sec. 2.2) which was considered that simplest type of heterogeneous media. In the case of absolute optical property recovery (Sec. 3.1) the significant result was that bottom-layer hemoglobin recovery is feasible, but scattering measurements are dominated by a top-layer. When examining dynamic changes in terms of sensitivities (Sec. 3.2) the main result was to be weary when measuring lipid dynamics due to potential cross-talk.

Overall, these simulation, analysis, and evaluation methods provide valuable guidance when interpreting data measured from a heterogeneous medium. While these results are specific to the model within this work, these types of simulations may be repeated or applied for other instruments and model media. Thus, this work is not only specific to this model but, also may be used as guidance on how other systems may be modeled to determine how heterogeneous media affect said system’s measurements.

## Figures and Tables

**Table 1.** Actual and recovered absorption parameters for baseline two-layer medium with a top layer thickness (*z*_Top_) of 5 mm.

	*T* (*μ*M)	*S* (%)	*W* (%)	*L* (%)	*μ*_*a*_ (650 nm) (1/mm)	*μ*_*a*_ (775 nm) (1/mm)	*μ*_*a*_ (900 nm) (1/mm)	*μ*_*a*_ (1024 nm) (1/mm)
Top actual	12	83	5	70	0.003	0.003	0.007	0.007
Bottom actual	120	67	90	20	0.042	0.026	0.034	0.042
Recovered	94	69	48	30	0.027	0.020	0.027	0.030

*Note*: Acronyms and variables: Total-hemoglobin (*T*), oxygen Saturation (*S*), Water (*W*), Lipid (*L*), and absorption coefficient (*μ*_*a*_).

**Table 2.** Actual and recovered scattering parameters for baseline two-layer medium with a top layer thickness (*z*_Top_) of 5 mm.

	$\mu_{s}^{\prime }(830\;\text{nm})$ (1/mm)	*b*	$\mu_{s}^{\prime }(690\;\text{nm})$ (1/mm)
Top actual	0.60	0.1	0.61
Bottom actual	0.40	1.5	0.53
Recovered	0.42	0.9	0.50

*Note*: Acronyms and variables: Reduced scattering coefficient $\left(\mu_{s}^{\prime }\right)$ and scattering power law exponent (*b*).

**Table 3.** Co-sensitivities for a two-layer medium with the baseline optical properties of Tables 1 and 2.

	*δT*/*δT*_lay_	*δS*/*δS*_lay_	*δW*/*δW*_lay_	*δL*/*δL*_lay_
Top	0.33	0.058	0.48	0.31
Bottom	0.62	0.63	0.41	0.58

*Note*: Acronyms and variables: Total-hemoglobin (*T*), oxygen Saturation (*S*), Water (*W*), Lipid (*L*).

**Table 4.** All sensitivities for a two-layer medium with the baseline optical properties of Tables 1 and 2.

	*δT* (*μ*M)	*δS*	*δW*	*δL*
*δT*_Top_ (*μ*M)	0.33	0.00010 *μ*M^−1^	0.00092 *μ*M^−1^	−0.0011 *μ*M^−1^
*δS*_Top_	−0.048 *μ*M	0.058	0.0017	−0.020
*δW*_Top_	−1.0 *μ*M	−0.0077	0.48	−0.21
*δL*_Top_	0.058 *μ*M	−0.00085	0.0074	0.31
*δT*_Bottom_ (*μ*M)	0.62	0.00043 *μ*M^−1^	−0.0011 *μ*M^−1^	−0.00016 *μ*M^−1^
*δS*_Bottom_	0.48 *μ*M	0.63	−0.036	0.16
*δW*_Bottom_	2.0 *μ*M	0.010	0.41	0.17
*δL*_Bottom_	0.20 *μ*M	0.0014	−0.0089	0.58

*Note*: Acronyms and variables: Total-hemoglobin (*T*), oxygen Saturation (*S*), Water (*W*), Lipid (*L*).

*Note* 1: Each element is the value of a numerical partial derivative with numerator corresponding to the top row labels and denominator corresponding to the left column labels.

*Note* 2: Co-sensitivities are diagonal elements of the top and bottom sub-tables and are restated in Table 3, off-diagonal elements are cross-sensitivities.

*Note* 3: All values are fractions none are represented in percent.

## Footnotes

a The scattering amplitude is expressed as the value of the reduced scattering coefficient $\left(\mu_{s}^{\prime }\right)$ at a single optical wavelength (*λ*).

b Total-hemoglobin (*T*) and oxygen Saturation (*S*) have more spectral meaning compared to Oxy-hemoglobin (*O*) and Deoxyhemoglobin (*D*).

c The full spectral simulation accounted for all 749 CW and two FD optical wavelength (*λs*).

d The extrapolated reduced scattering coefficient $\left(\mu_{s}^{\prime }\right)$ was retrieved from Eqs. (3) to (7) and is referred to as $\mu_{s}^{\prime }\left(\lambda_{\text{CW}}\right)$.

e The effective homogeneous properties recovered are also dependent on the specific instrument simulated, particularly the source-detector distance (*ρs*) and optical wavelengths (*λs*).

f This reduced scattering coefficient $\left(\mu_{s}^{\prime }\right)$ at a single optical wavelength (*λ*) is the scattering amplitude, and the single *λ* used is 830 nm.

g Inputs to the two-layer forward model are considered independent variables.

h The small amount used to calculate the numerical derivative was the baseline value of the parameter multiplied by 10^−6^.

i Dependent variables are the outputs of the homogeneous inverse model.

j An example of a co-sensitivity would be between top layer Total-hemoglobin (*T*_Top_) and Total-hemoglobin (*T*) or *δT*/*δT*_top_.

k An example of a cross-sensitivity would be between top layer Water (*W*_Top_) and Total-hemoglobin (*T*) or *δT*/*δT*_top_.

l Parameters were varied without co-variation, meaning while one input parameter varied all other input parameters were kept fixed to their baseline value.

m Determination of how close a recovered value is to the actual is done by comparing the solid lines to the dashed or dotted lines.

n This result can only be said to be true for the instrument simulated and baseline parameters used.

o Simulating no changes in scattering in the forward model and assuming no changes in scattering when recovering absorption changes.

p Sensitivities between like parameters are dubbed co-sensitivities, between recovered *T* and *T*_Top_ for example.

q Sensitivities between dis-similar parameters are cross-sensitivities, for example between recovered *T* and *W*_Top_.

r Little cross-sensitivity except in the case of recovered *L* changes, which typically is assumed to not have dynamics.

s The range of physically possible scattering power law exponent (*b*) is between zero and four.

## Appendix A

The following diffusion theory derived expressions support the material in the main article and allow the reader to recreate the simulations. To emphasize that the main independent variables are the source-detector distance (*ρ*), the absorption coefficient (*μ*_*a*_), the reduced scattering coefficient $\left(\mu_{s}^{\prime }\right)$, and the top layer thickness (*z*_Top_), the dependent variables in these expressions are shown as functions of them. Other variables are in fact independent but are not included in this notation since they are not varied within this work.^t^ Furthermore, for brevity, this function notation is only used on the left-hand-side of the following expressions.

### A.1. Expression for two-layer reflectance

To express the two-layer (cylinder)^7^ complex reflectance, first, the following values are defined. The speed of light within the medium (*v*):

(A.1)
v=c/n,

where *c* is the speed of light in vacuum (2.99792458 × 10^11^ mm/s) and *n*is the index of refraction within the medium. The angular modulation frequency (*ω*):

(A.2)
$$\omega =2\pi f_{\text{mod}}$$

where *f*_mod_ is the modulation frequency (140.625 MHz for FD and 0 Hz for CW). The diffusion coefficient (D) is

(A.3)
$$D\left(\mu_{s,\text{top}/\text{bot}}^{\prime }\right)=\frac{1}{3\mu_{s,\text{top}/\text{bot}}^{\prime }},$$

where $\mu_{s}^{\prime }$ is the reduced scattering coefficient. The approximate depth of the isotropic source (*z*_0_):

(A.4)
$$z_{0}\left(\mu_{s,\text{top}}^{\prime }\right)=\frac{1}{\mu_{s,\text{top}}^{\prime }}.$$

The approximate extrapolated boundary height (*z*_*b*_):

(A.5)
$$z_{b}\left(\mu_{s,\text{top}}^{\prime }\right)=2AD_{\text{top}},$$

where *n* is the index of refraction of the medium (assumed homogeneous here) and *A* is the index of refraction mismatch parameter.^18^

Next, various coefficients are defined as a function of the *k*th zero of the 0th-order Bessel function of the first kind. First, the optical attenuation parameter $\left({\tilde{a}}_{k}\right)$:

(A.6)
$${\tilde{a}}_{k}\left(\mu_{a,\text{top}/\text{bot}},\mu_{s,\text{top}/\text{bot}}^{\prime }\right)=\sqrt{\frac{\mu_{a,\text{top}/\text{bot}}}{D_{\text{top}/\text{bot}}}+{\left(\frac{j_{0,k}}{B}\right)}^{2}+\frac{\omega }{vD_{\text{top}/\text{bot}}}}i,$$

where *μ*_*a*_ is the absorption coefficient, *j*_0,*k*_ the *k*th zero of the 0th-order Bessel function of the first kind, *B* is the radius of the two-layer cylinder (for which the source is in the center; assumed to be 150 mm for this work), and *i* is the imaginary unit. Then, the source-detector distance parameter (*Q*_*k*_):

(A.7)
$$Q_{k}(\rho )=\frac{J_{0}\left(\frac{\rho j_{0,k}}{B}\right)}{J_{1}{\left(j_{0,k}\right)}^{2}},$$

where *J*_*ν*_ is the *ν*th-order Bessel function of the first kind and *ρ* is the source-detector distance.

Now, the reflectance for the case when the isotropic source is in the first layer $\left({\tilde{R}}_{1}\right)$ can be expressed:

(A.8)
$${\tilde{R}}_{1}\left(\rho ,\mu_{a,\text{top}},\mu_{s,\text{top}}^{\prime },z_{\text{top}},\mu_{a,\text{bot}},\mu_{s,\text{bot}}^{\prime }\right)\;=\frac{1}{\pi B^{2}}\sum_{k=1}^{\infty }\{[\frac{e^{-{\tilde{a}}_{\text{top},k}z_{0}}+e^{-{\tilde{a}}_{\text{top},k}\left(z_{0}+2z_{b}\right)}}{2}\;+\frac{\text{cosh}\left({\tilde{a}}_{\text{top},k}z_{0}\right)\times \text{sinh}\left({\tilde{a}}_{\text{top},k}\left(z_{0}+2z_{b}\right)\right)}{e^{{\tilde{a}}_{\text{top},k}\left(z_{\text{top}}+z_{b}\right)}}\;\times \left(D_{\text{top}}{\tilde{a}}_{\text{top},k}-D_{\text{bot}}{\tilde{a}}_{\text{bot},k}\right)\;/(D_{\text{top}}{\tilde{a}}_{\text{top},k}\text{cosh}\left({\tilde{a}}_{\text{top},k}\left(z_{\text{top}}+z_{b}\right)\right)\;+D_{\text{bot}}{\tilde{a}}_{\text{bot},k}\text{sinh}\left({\tilde{a}}_{\text{top},k}\left(z_{\text{top}}+z_{b}\right)\right))]Q_{k}\},$$

where *z*_top_ is the thickness of the top layer.^u^

To express the reflectance when the isotropic source is in the second layer $\left({\tilde{R}}_{2}\right)$ additional coefficients are defined to simplify the expression:

(A.9)
$${\tilde{\alpha }}_{1,k}\left(\mu_{a,\text{top}},\mu_{s,\text{top}}^{\prime }\right)=e^{-{\tilde{a}}_{\text{top},k}z_{b}},$$

(A.10)
$${\tilde{\alpha }}_{2,k}\left(\mu_{a,\text{top}},\mu_{s,\text{top}}^{\prime },z_{\text{top}}\right)=n^{2}e^{{\tilde{a}}_{\text{top},k}z_{\text{top}}},$$

(A.11)
$${\tilde{\alpha }}_{3,k}\left(\mu_{a,\text{top}},\mu_{s,\text{top}}^{\prime },z_{\text{top}}\right)={\tilde{a}}_{\text{top},k}D_{\text{top}}e^{{\tilde{a}}_{\text{top},k}z_{\text{top}}},$$

(A.12)
$${\tilde{\beta }}_{1,k}\left(\mu_{a,\text{top}},\mu_{s,\text{top}}^{\prime }\right)=e^{{\tilde{a}}_{\text{top},k}z_{b}},$$

(A.13)
$${\tilde{\beta }}_{2,k}\left(\mu_{a,\text{top}},\mu_{s,\text{top}}^{\prime },z_{\text{top}}\right)=n^{2}e^{-{\tilde{a}}_{\text{top},k}z_{\text{top}}},$$

(A.14)
$${\tilde{\beta }}_{3,k}\left(\mu_{a,\text{top}},\mu_{s,\text{top}}^{\prime },z_{\text{top}}\right)=-{\tilde{a}}_{\text{top},k}D_{\text{top}}e^{-{\tilde{a}}_{\text{top},k}z_{\text{top}}},$$

(A.15)
$${\tilde{\gamma }}_{2,k}\left(z_{\text{top}},\mu_{a,\text{bot}},\mu_{s,\text{bot}}^{\prime }\right)=-n^{2}e^{-{\tilde{a}}_{\text{bot},k}z_{\text{top}}},$$

(A.16)
$${\tilde{\gamma }}_{3,k}\left(z_{\text{top}},\mu_{a,\text{bot}},\mu_{s,\text{bot}}^{\prime }\right)={\tilde{a}}_{\text{bot},k}D_{\text{bot}}e^{-{\tilde{a}}_{\text{bot},k}z_{\text{top}}},$$

(A.17)
$${\tilde{\zeta }}_{2,k}\left(\mu_{s,\text{top}}^{\prime },z_{\text{top}},\mu_{a,\text{bot}},\mu_{s,\text{bot}}^{\prime }\right)=-\frac{n^{2}e^{-{\tilde{a}}_{\text{bot},k}\left(z_{\text{top}}-z_{0}\right)}}{2{\tilde{a}}_{\text{bot},k}D_{\text{bot}}},$$

(A.18)
$${\tilde{\zeta }}_{3,k}\left(\mu_{s,\text{top}}^{\prime },z_{\text{top}},\mu_{a,\text{bot}},\mu_{s,\text{bot}}^{\prime }\right)=-\frac{e^{{\tilde{a}}_{\text{bot},k}\left(z_{\text{top}}-z_{0}\right)}}{2}.$$

Then combinations of these coefficients are defined.

(A.19)
$${\tilde{\Xi }}_{k}\left(\mu_{a,\text{top}},\mu_{s,\text{top}}^{\prime },z_{\text{top}},\mu_{a,\text{bot}},\mu_{s,\text{bot}}^{\prime }\right)\;={\tilde{\alpha }}_{1,k}{\tilde{\beta }}_{2,k}{\tilde{\gamma }}_{3,k}-{\tilde{\alpha }}_{1,k}{\tilde{\beta }}_{3,k}{\tilde{\gamma }}_{2,k}\;-{\tilde{\alpha }}_{2,k}{\tilde{\beta }}_{1,k}{\tilde{\gamma }}_{3,k}+{\tilde{\alpha }}_{3,k}{\tilde{\beta }}_{1,k}{\tilde{\gamma }}_{2,k},$$

(A.20)
$${\tilde{\Upsilon }}_{k}\left(\mu_{a,\text{top}},\mu_{s,\text{top}}^{\prime },z_{\text{top}},\mu_{a,\text{bot}},\mu_{s,\text{bot}}^{\prime }\right)\;=-\frac{{\tilde{\beta }}_{1,k}\left({\tilde{\gamma }}_{2,k}{\tilde{\zeta }}_{3,k}-{\tilde{\gamma }}_{3,k}{\tilde{\zeta }}_{2,k}\right)}{{\tilde{\Xi }}_{k}},$$

(A.21)
$${\tilde{\Psi }}_{k}\left(\mu_{a,\text{top}},\mu_{s,\text{top}}^{\prime },z_{\text{top}},\mu_{a,\text{bot}},\mu_{s,\text{bot}}^{\prime }\right)\;=\frac{{\tilde{\alpha }}_{1,k}\left({\tilde{\gamma }}_{2,k}{\tilde{\zeta }}_{3,k}-{\tilde{\gamma }}_{3,k}{\tilde{\zeta }}_{2,k}\right)}{{\tilde{\Xi }}_{k}}.$$

Given all these coefficients the reflectance when the isotropic source is in the second layer $\left({\tilde{R}}_{2}\right)$ can now be expressed:^*u*^

(A.22)
$${\tilde{R}}_{2}\left(\rho ,\mu_{a,\text{top}},\mu_{s,\text{top}}^{\prime },z_{\text{top}},\mu_{a,\text{bot}},\mu_{s,\text{bot}}^{\prime }\right)\;=\frac{1}{\pi B^{2}}\sum_{k=1}^{\infty }\left\{\left[D_{\text{top}}{\tilde{a}}_{\text{top},k}\left({\tilde{\Upsilon }}_{k}-{\tilde{\Psi }}_{k}\right)\right]Q_{k}\right\}.$$

Finally, the piece-wise expression for the complex reflectance from a two-layer medium may be expressed $\left(\tilde{R}\right)$:

(A.23)
$$\tilde{R}\left(\rho ,\mu_{a,\text{top}},\mu_{s,\text{top}}^{\prime },z_{\text{top}},\mu_{a,\text{bot}},\mu_{s,\text{bot}}^{\prime }\right)\;=\{\begin{array}{ll} {\tilde{R}}_{1}, & z_{0}<z_{\text{top}},\\ {\tilde{R}}_{2}, & z_{0}\geq z_{\text{top}}. \end{array}$$

### A.2. Recovery of absolute optical properties

#### A.2.1. Expression for semi-infinite reflectance

The semi-infinite complex reflectance $\left(\tilde{R}\right)$ can be expressed as a function of the source-detector distance (*ρ*) and the complex effective attenuation coefficient ${\tilde{\mu }}_{\text{eff}}$:^v,1,8^

(A.24)
$$\tilde{R}\left(\rho ,\mu_{a},\mu_{s}^{\prime }\right)\;=\frac{1}{4\pi }\left({\tilde{C}}_{1}e^{-{\tilde{\mu }}_{\text{eff}}r_{1}}+{\tilde{C}}_{2}e^{-{\tilde{\mu }}_{\text{eff}}r_{2}}\right),$$

where ${\tilde{C}}_{1}$, ${\tilde{C}}_{2}$, *r*_1_, and *r*_2_ are parameters defined as follows:

(A.25)
$${\tilde{C}}_{1}\left(\rho ,\mu_{a},\mu_{s}^{\prime }\right)=z_{0}\left(\frac{1+{\tilde{\mu }}_{\text{eff}}r_{1}}{r_{1}^{3}}\right),$$

(A.26)
$${\tilde{C}}_{2}\left(\rho ,\mu_{a},\mu_{s}^{\prime }\right)=-z_{0}^{\prime }\left(\frac{1+{\tilde{\mu }}_{\text{eff}}r_{2}}{r_{2}^{3}}\right),$$

(A.27)
$$r_{1}\left(\rho ,\mu_{s}^{\prime }\right)=\sqrt{\rho^{2}+z_{0}^{2}},$$

(A.28)
$$r_{2}\left(\rho ,\mu_{a},\mu_{s}^{\prime }\right)=\sqrt{\rho^{2}+z_{0}^{\prime^{2}}},$$

(A.29)
$$z_{0}\left(\mu_{s}^{\prime }\right)=\frac{1}{\mu_{s}^{\prime }},$$

(A.30)
$$z_{0}^{\prime }\left(\mu_{a},\mu_{s}^{\prime }\right)=-z_{0}+2z_{b},$$

(A.31)
$$z_{b}\left(\mu_{a},\mu_{s}^{\prime }\right)=\frac{2A}{3\left(\mu_{s}^{\prime }+\mu_{a}\right)},$$

where $\mu_{s}^{\prime }$ is the reduced scattering coefficient, *μ*_*a*_ is the absorption coefficient, *n* is the index of refraction of the medium, and *A* is the index of refraction mismatch parameter.^18^ In the Continuous-Wave (CW) case all complex variables become real and the expressions are still valid.^w^

#### A.2.2. Inversion of semi-infinite reflectance

Given that the measurement method focuses on measuring complex $\left(\tilde{R}\right)$ or real (*R*) reflectance versus source-detector distance (*ρ*),^x^ Eq. (A.24) is rearranged to find a linear relationship between the measured reflectance and the effective attenuation coefficient $\left({\tilde{\mu }}_{\text{eff}}\right)$ across *ρ*:

(A.32)
$$\text{ln}\left[(4\pi \tilde{R})/\left({\tilde{C}}_{1,m-1}+{\tilde{C}}_{2,m-1}e^{{\tilde{\mu }}_{\text{eff},m-1}\left(r_{1,m-1}-r_{2,m-1}\right)}\right)\right]\;=-{\tilde{\mu }}_{\text{eff},m}r_{1,m-1}.$$

Here the notation of dependent variables being a function of independent is dropped. With an initial guess for ${\tilde{\mu }}_{\text{eff}}$ the slope of the left-hand-side of Eq. (A.32) versus *r*_1_ can be fit^y^ to find an updated estimate of ${\tilde{\mu }}_{\text{eff}}$. This procedure is repeated iteratively, where *m* is the iteration number, until ${\tilde{\mu }}_{\text{eff}}$ converges. The initial guess was chosen using previously described methods which make further assumptions to achieve simpler expressions.^z,19^ For CW data the same procedure is used, only with the complex variables replaced with the real ones and the real effective attenuation coefficient (*μ*_eff_) as the output instead of the complex one.
